# The pocket-creation method facilitates gastric endoscopic submucosal dissection and overcomes challenging situations

**DOI:** 10.1016/j.vgie.2021.05.001

**Published:** 2021-05-28

**Authors:** Masafumi Kitamura, Yoshimasa Miura, Satoshi Shinozaki, Hironori Yamamoto

**Affiliations:** 1 Department of Medicine, Division of Gastroenterology, Jichi Medical University, Shimotsuke, Japan; 2 Shinozaki Medical Clinic, Utsunomiya, Japan

**Keywords:** ESD, endoscopic submucosal dissection, PCM, pocket-creation method, ST-hood, small-caliber tip transparent hood

## Abstract

Video 1 Demonstration of gastric endoscopic submucosal dissection using the pocket-creation method at the angle along the lesser curvature and fornix.

Demonstration of gastric endoscopic submucosal dissection using the pocket-creation method at the angle along the lesser curvature and fornix.

## Introduction

Endoscopic submucosal dissection (ESD) is the criterion standard for resection of gastric superficial tumors. However, the stomach has a wide lumen that makes gastric ESD difficult in some locations. In difficult locations in the stomach, sometimes a distant or a vertical approach toward the muscularis is unavoidable and results in lengthy procedure times, the occurrence of adverse events, and a low-quality resected specimen. Unlike colorectal ESD, changing the patient’s position during the procedure is very difficult during gastric ESD when the patient is under conscious sedation. Use of the traction method and a multibending endoscope are viable options to facilitate ESD in difficult circumstances.^1^^,^^2^

The pocket-creation method (PCM) is a novel strategy to achieve safe and high-quality ESD.^3^ Initially, it was developed for resecting large sessile colorectal polyps with dense submucosal fibrosis, but it has been applied to duodenal and colorectal ESD in various locations. The PCM has become recognized as a universal strategy for ESD throughout the alimentary tract. The PCM has 4 representative advantages: (1) the injected solution is not dispersed, owing to a minimal incision; (2) both traction and countertraction are obtained simultaneously because the transparent hood stretches the submucosal tissue in the limited space; (3) a vertical approach toward the muscularis can be changed to a tangential approach; and (4) the influence of cardiopulmonary movement is diminished because of synchronization between the endoscope and the pocket.^4^

Even in the wide lumen of the stomach, a stable antegrade approach can be obtained using the PCM. We here present 2 representative cases of gastric superficial lesions resected by ESD with the PCM (Video 1, available online at www.VideoGIE.org).

## Procedure sequence of PCM

**Figure 1:**
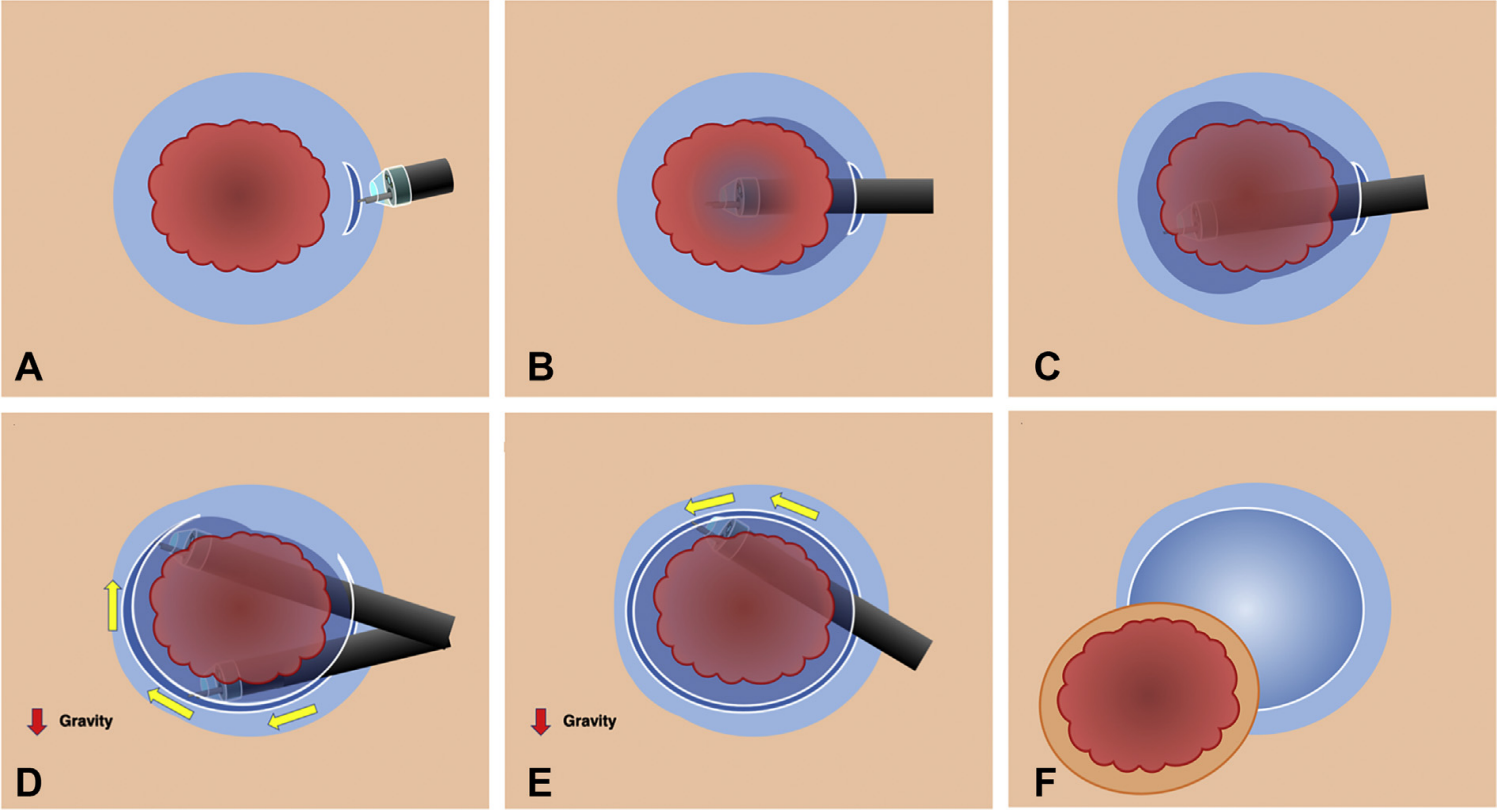
Schematic of the pocket-creation method. **A,** A minimal mucosal incision is made at least 5 mm from the edge of the tumor. **B and C,** A submucosal pocket is created under the tumor without a circumferential incision. **D,** The pocket is opened in a step-by-step manner from the gravity side. **E,** The remaining area is dissected. **F,** An en bloc resection is accomplished.

**Figure 2:**
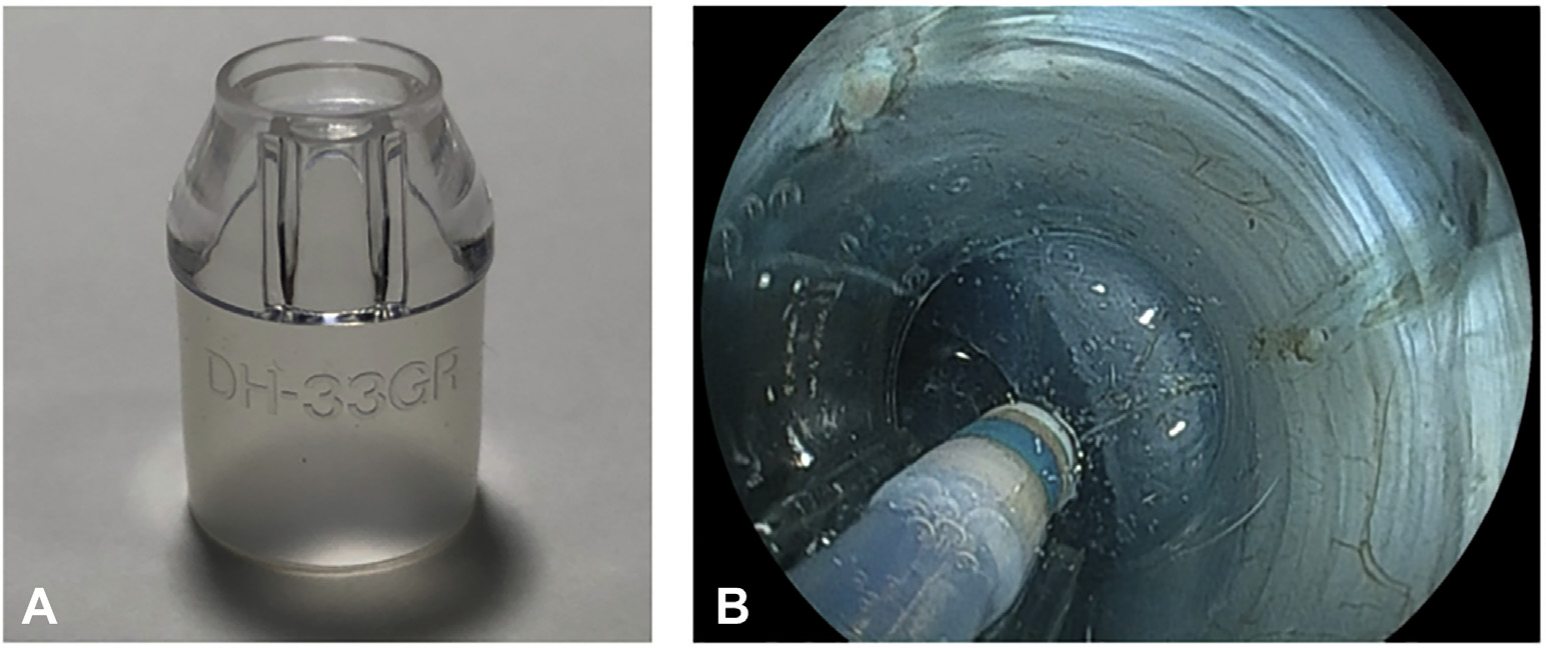
A small-caliber-tip transparent (ST) hood. **A,** The ST-hood is tapered and has a narrow orifice. **B,** The ST-hood provides effective traction and leads the knife to the center of endoscopic vision.

First, a minimal mucosal incision is made at least 5 mm from the edge of the tumor (Fig. 1). Second, prudent submucosal dissection is required to get the tip of the endoscope into the pocket, and the pocket then is extended by the tip of the hood without a circumferential incision to maintain both traction and countertraction and prevent dispersion of the injected solution. Third, after completion of submucosal dissection under the tumor, the pocket is completely opened from the gravity side. In gastric ESD with PCM, aspirating as much gas as possible from the gastric lumen is one of the most important tips for a successful procedure. Decreasing the size of the gastric lumen by aspirating gas can improve the maneuverability of the endoscope and facilitate a tangential approach. A small-caliber-tip transparent (ST) hood (DH-15GR or DH-33GR; Fujifilm Corp, Tokyo, Japan) is essential to perform the PCM (Fig. 2). The ST-hood is tapered and has a narrow orifice that facilitates entering the pocket and provides traction and countertraction. Furthermore, the ST-hood has a groove that leads the knife to the center of endoscopic vision, which is important in selecting the dissection level in the submucosal layer. The dissection level should be just above the muscularis to obtain a high-quality specimen with a thick submucosal layer.

## Case presentations

### Patient 1

**Figure 3:**
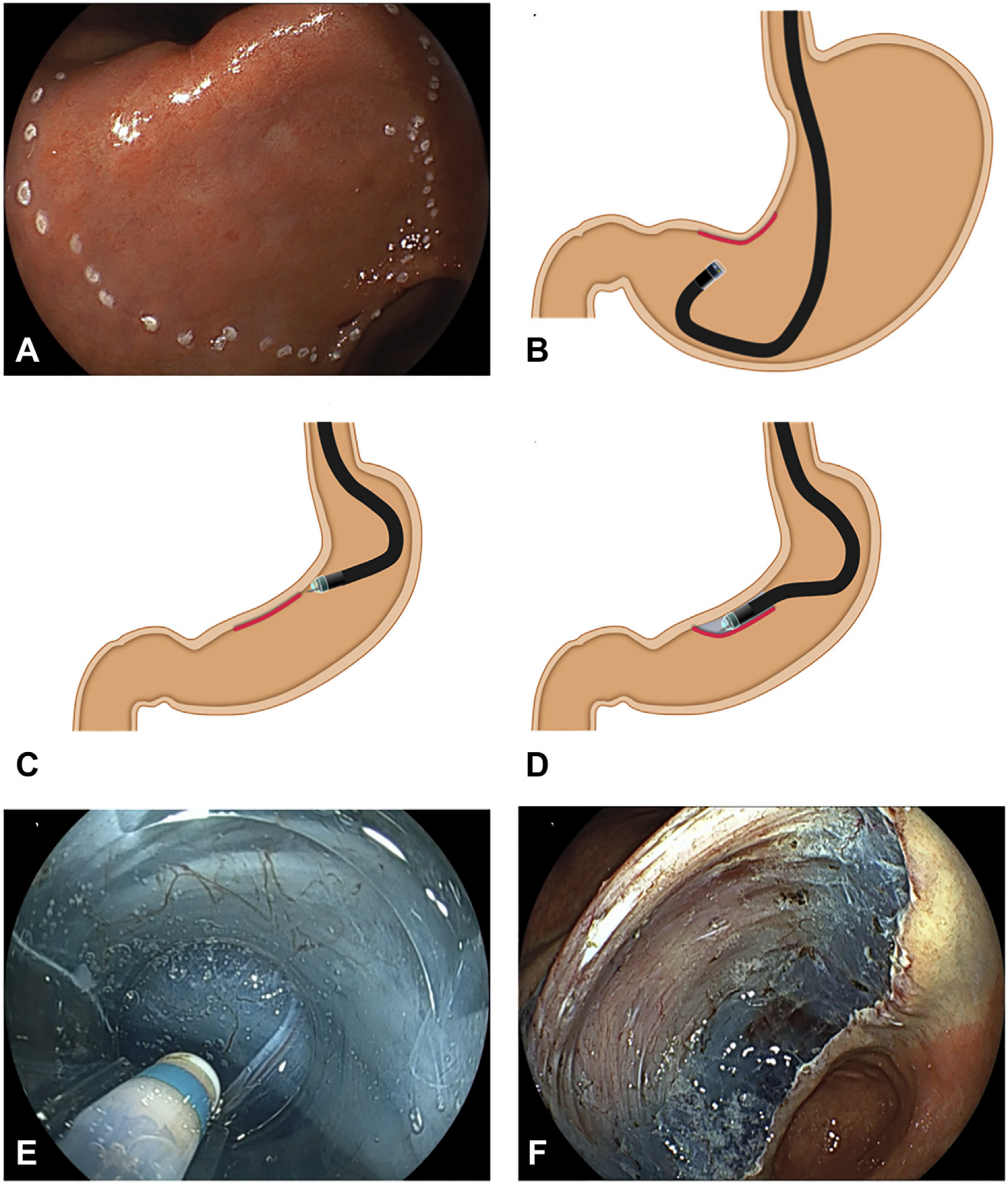
Patient 1. **A,** A 70-mm flat lesion at the angle along the lesser curvature of the stomach. **B,** In the inflated stomach, a vertical approach is inevitable in retroflexion. **C,** With minimal inflation and without a redundant loop of the endoscope, the pocket-creation method enabled an antegrade, tangential approach. **D,** After the pocket is entered, submucosal dissection progresses with an antegrade approach from the angle to the antrum. **E,** Clear visualization of the submucosal layer in the pocket using a transparent hood enables selection of the dissection level by allowing the operator to recognize blood vessels and the muscularis. **F,** The mucosal defect after endoscopic resection.

A 70-mm flat lesion was located at the angle along the lesser curvature (Fig. 3A). In the inflated stomach, an approach to such a lesion was difficult, and a vertical approach is inevitable in retroflexion (Fig. 3B). With minimal inflation and without a redundant loop of the endoscope, the PCM enabled an antegrade, tangential approach (Fig. 3C). After creation of a minimal incision and pocket formation, the tip of the endoscope was stabilized in the pocket. Stabilization enables submucosal dissection from the angle to the antrum (Fig. 3D). Clear visualization of the submucosal layer in the pocket using a transparent hood enables selection of the dissection level by allowing the surgeon to recognize blood vessels and the muscularis (Fig. 3E). En bloc resection is uneventfully accomplished (Fig. 3F).

### Patient 2

**Figure 4:**
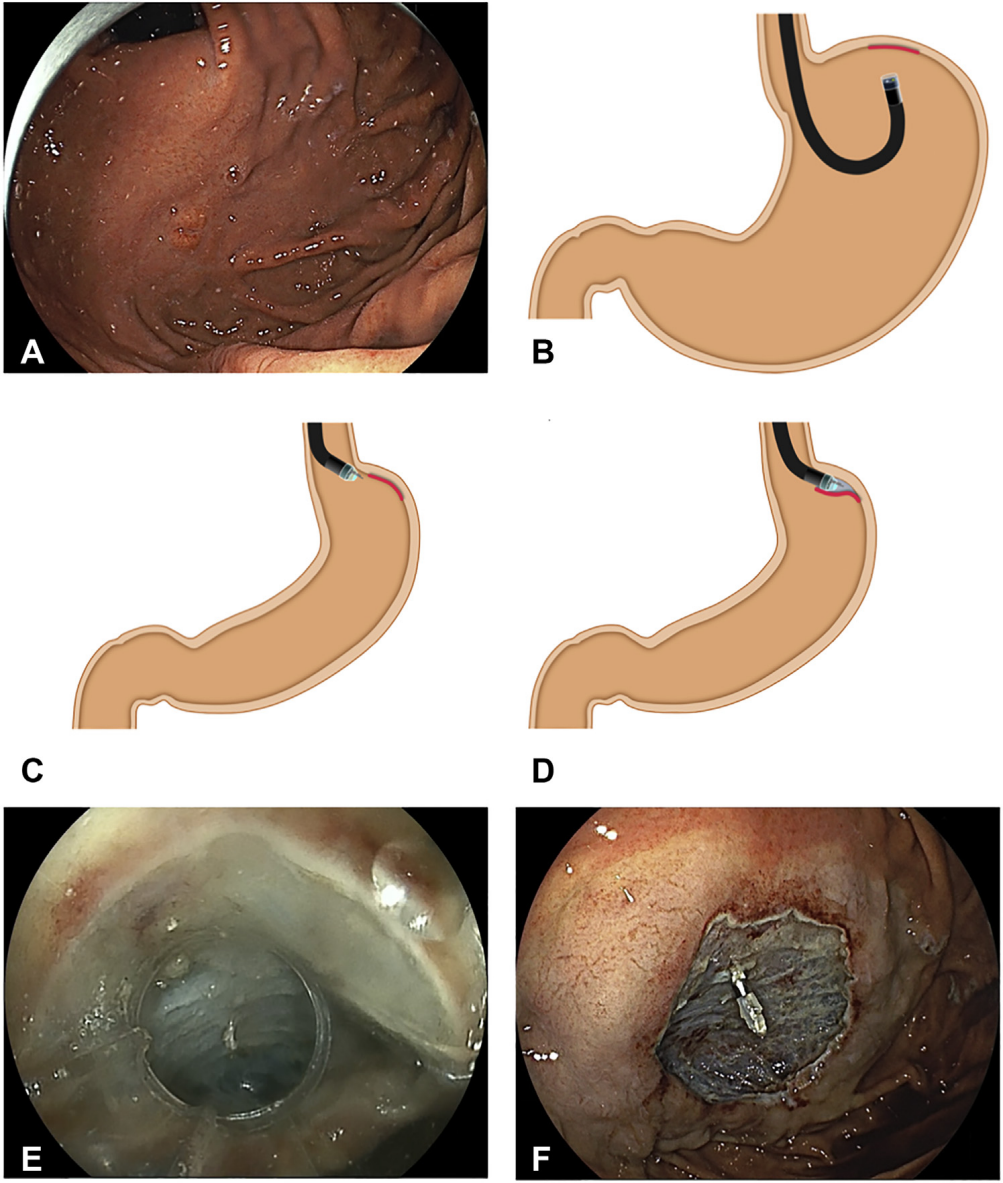
Patient 2. **A,** A 12-mm elevated lesion is located at the fornix. **B,** In the inflated stomach, a distant vertical approach is inevitable in retroflexion. **C,** With minimal inflation, the pocket-creation method enabled an antegrade, tangential approach even at the fornix. **D,** After pocket formation, submucosal dissection under the lesion can be performed with an antegrade approach. **E,** The pocket created after submucosal dissection under the lesion is completed. **F,** The mucosal defect after endoscopic resection.

A 12-mm elevated lesion was located at the fornix (Fig. 4A). In the inflated stomach, a distant and vertical approach is inevitable in retroflexion (Fig. 4B). With minimal inflation by aspirating gas, the PCM enabled an antegrade, tangential approach even at the fornix (Fig. 4C). After creation of a minimal incision and pocket formation, submucosal dissection under the lesion can be performed with an antegrade approach (Fig. 4D and E). In the pocket, the same clear visualization is obtained regardless of the location of the lesion. En bloc resection is uneventfully accomplished (Fig. 4F).

## Discussion

The greatest advantage of the PCM in gastric ESD is stabilization in the pocket at any location in the stomach. When the conventional method is used, the difficulty of gastric ESD is strongly influenced by the location, unlike in other alimentary organs with narrow lumens, such as the esophagus and colorectum. To resect lesions located along the lesser curvature at the angle and fornix, retroflexion is generally necessary using the conventional method. However, retroflexion can make approaching the lesion difficult, and sometimes a distant approach is unavoidable. With a distant approach, endoscopists have to protrude the sheath of the endoknife in the gastric lumen, leading to rough and unstable movements. In such a situation, submucosal dissection without visualization of blood vessels or the muscularis increases the risk of bleeding and perforation. Even if the endoscopic approach is straightforward, a vertical approach is inevitable at the gastric angle and fornix. The vertical approach makes dissection difficult and increases the risk of damaging the muscularis. The PCM enables an antegrade approach regardless of the location and without need for special devices such as a multibending endoscope or traction tools.

There are some reports about the usefulness of the PCM in overcoming other challenging situations, such as complicated anatomy and submucosal fibrosis. A gastric neoplasm involving the pyloric ring is among the difficult situations encountered in ESD of gastric lesions. The submucosa under the tumor must be dissected, getting over the firm and high muscularis at the pyloric ring; then the endoscope approach becomes vertical to the sphincter and the tip of the endoscope goes into the lesion, resulting in damage to the specimen. In addition, severe submucosal fibrosis leads a low en bloc resection rate, lengthy procedure time, and increased adverse events.^5^ The PCM facilitates gastric ESD even in these difficult circumstances.^6^^,^^7^

In the pocket-opening procedure in the final stage of PCM, loss of stabilization creates a difficult situation in some locations. We always deal with this by starting to open the pocket from the gravity side in a step-by-step manner.^8^ If this does not work, traction methods or saline-immersion therapeutic endoscopy may help to open the pocket.^9^^,^^10^

In this video, we illustrated 2 cases that would be difficult using the conventional method because of their location. However, the PCM is not only useful for conquering lesions at difficult locations but also for resecting routine lesions more easily. The PCM is a simple technique to achieve safe ESD, facilitating a tangential approach that enables retrieval of a specimen with a thick submucosal layer by selecting the dissection level. Although gastric ESD is widely performed at many institutions, endoscopists struggle to resect superficial gastric lesions under difficult conditions. Gastric ESD using the PCM can be performed with a uniform strategy, regardless of the location of the lesion, and it is more straightforward for the operator than conventional methods. We believe that the PCM is a universal strategy for gastric ESD.

## Supplementary data

Video 1 Demonstration of gastric endoscopic submucosal dissection using the pocket-creation method at the angle along the lesser curvature and fornix.
